# Superoxide stability for reversible Na-O_2_ electrochemistry

**DOI:** 10.1038/s41598-017-17745-9

**Published:** 2017-12-15

**Authors:** V. S. Dilimon, Chihyun Hwang, Yoon-Gyo Cho, Juchan Yang, Hee-Dae Lim, Kisuk Kang, Seok Ju Kang, Hyun-Kon Song

**Affiliations:** 1 School of Energy and Chemical Engineering, UNIST, Ulsan, 44919 Korea; 2 Department of Materials Science and Engineering, Research Institute of Advanced Materials (RIAM), Seoul National University, Seoul, 08826 Korea

## Abstract

Stabilizing superoxide (O_2_^−^) is one of the key issues of sodium-air batteries because the superoxide-based discharge product (NaO_2_) is more reversibly oxidized to oxygen when compared with peroxide (O_2_^2−^) and oxide (O^2−^). Reversibly outstanding performances of sodium-oxygen batteries have been realized with the superoxide discharge product (NaO_2_) even if sodium peroxide (Na_2_O_2_) have been also known as the discharge products. Here we report that the Lewis basicity of anions of sodium salts as well as solvent molecules, both quantitatively represented by donor numbers (DNs), determines the superoxide stability and resultantly the reversibility of sodium-oxygen batteries. A DN map of superoxide stability was presented as a selection guide of salt/solvent pair. Based on sodium triflate (CF_3_SO_3_^−^)/dimethyl sulfoxide (DMSO) as a high-DN-pair electrolyte system, sodium *ion* oxygen batteries were constructed. Pre-sodiated antimony (Sb) was used as an anode during discharge instead of sodium metal because DMSO is reacted with the metal. The superoxide stability supported by the high DN anion/solvent pair (${{\rm{CF}}}_{3}{{\rm{SO}}}_{3}$ ^–^/DMSO) allowed more reversible operation of the sodium *ion* oxygen batteries.

## Introduction

Lithium metal-oxygen (Li-O_2_) batteries have the highest theoretical specific energy (3450 Wh kg^−1^) than any other reported battery systems^1–3^. However, poor rechargeability and low energy efficiency (~60%) due to high polarization during charging are the major concerns of Li-O_2_ batteries^1–4^. Hartmann *et al*. demonstrated that just substituting metallic Na for Li in metal-air batteries results in extremely low polarization and higher energy efficiency (~90%) even without the use of any catalysts^5^. Even if the theoretical specific energy of Na-O_2_ batteries is lower (1605 and 1100 Wh kg^−1^ respectively for Na_2_O_2_ and NaO_2_ discharge products) than that of Li-O_2_ batteries, the value is still higher than any other metal-air batteries and lithium-ion batteries^3^. Further, the capacities of Na-O_2_ batteries were significantly higher at lower current densities than similarly discharged Li-O_2_ batteries^4,5^. The Na-O_2_ batteries would have more practical significance as the available Li resources were depleted^3^. Sodium is the earth’s sixth most abundant element.

As in Li-O_2_ cells, the major solid discharge product of Na-O_2_ cells has been identified as peroxide salt (Na_2_O_2_; similarly Li_2_O_2_ in Li-O_2_ cells)^6–10^ before Hartmann *et al*.’s work^5^. Superoxide ion (O_2_^−^), which is the first product of oxygen reduction reactions (ORRs), was considered unstable in electrolyte and to be converted readily to peroxide ion (O_2_^2−^). Electrolytes used in the Na-O_2_ cells reported to discharge solid peroxide includes: NaPF_6_ in EC:DMC^7^ (EC = ethylene carbonate; DMC = dimethyl carbonate) or DME (dimethoxy ethane)^8^; NaClO_4_ in TEGDME (tetraethylene glycol dimethyl ether)^9^; sodium triflate (NaSO_3_CF_3_) in an ionic liquid (1-ethyl-3-methyl imidazolium trifluoromethanesulfonate)^10^.

However, Hartmann *et al*. reported superoxide salt (NaO_2_) as the solid discharge product of ORR in their Na-O_2_ cells based on NaSO_3_CF_3_ in DEGDME (diethylene glycol dimethyl ether), showing significantly improved performances of Na-O_2_ batteries^5^. The superoxide (NaO_2_) is not thermodynamically more favored relative to peroxide (Na_2_O_2_) (E^0^ (Na_2_O_2_) = 2.33 V versus E^0^ (NaO_2_) = 2.27 V). Analogous Li-O_2_ electrochemistry leads only to Li_2_O_2_ formation^4^. Even though the reasons for the differences between Li-O_2_ and Na-O_2_ electrochemistry are still ambiguous, Hartmann *et al*. explained that the superoxide formation is kinetically feasible because smaller number of electron transfer (one electron) is involved in the process than in peroxide formation (two electrons). Later works also confirmed the superoxide discharge product in the same electrolyte^4,11–15^. The main appeal of the Hartmann’s Na-O_2_ cell was its very low overpotential during charging (less than 200 mV) which was 3–4 times lower than any other reported Li-O_2_ or Na-O_2_ cells^5^. The possible reason for this high performance was the higher electronic conductivity of NaO_2_^5,16^. In theoretical calculation, the major discharge product is controversial between superoxide and peroxide^16,17^. Superoxide formation might compete with peroxide formation so that operational condition such as discharge currents could determine the composition of discharge products^12,18^. NaO_2_ was more favored at higher current densities while Na_2_O_2_ was more favored at lower current densities^18^. The Na-O_2_ battery research is still in an infant stage. Therefore, a clear awareness of the factors determining the final discharge product is crucial for the practical development of Na-O_2_ battery.

By analyzing previous works on discharge products of Na-O_2_ electrochemistry, solvents and anions of sodium salts in electrolytes are expected as important factors to determine the final products or superoxide stabilization. Stability of superoxide in non-aqueous electrolytes containing alkali metal cations was explained by using acidity of the alkali metal cations and basicity (donor number, DN) of the non-aqueous solvents^19–21^. On the basis of Pearson’s hard soft acid base (HSAB) theory, Li^+^ and Na^+^ as hard Lewis acids show higher affinity for peroxide (O_2_^2−^) and oxide anions (O^2−^) which are hard Lewis bases. However, solvents of high basicity (high DN value) can solvate these alkali metal cations to produce soft cation complexes. The solvated cation (soft cation) can make a stable ion pair with O_2_^−^ (superoxide ion as a soft base) in solution. Unlike lithium superoxide which cannot have a stable existence as solid discharge product^22,23^, sodium superoxide is stable and it can crystallize on Na-O_2_ battery cathode^5,11,12,18^. It is important to note that the works of Hartmann *et al*. showing the superoxide formation were with a high DN solvent DEGDME (DN = ~24)^5,11–13,24^. On the contrary, peroxide and/or its hydrated form (Na_2_O_2_ and Na_2_O_2_∙2H_2_O) were formed in comparatively lower DN solvents, DME (DN = 20.0) and TEGDME (DN = 16.6), respectively^8,9,19^. Therefore, it looks clear that the solvents affect the superoxide stability and therefore determining discharge products.

However, the superoxide formation as the discharge product was reported even in the lower DN solvent such as DME in the presence of NaSO_3_CF_3_^4^.^14^^,^ On the other hand, the works reporting the superoxide formation have consistently used NaSO_3_CF_3_ as the supporting salt^4,5,11–13^. Therefore, it would be one of easy inferences that anions, in addition to solvent molecules, play a crucial role on stabilizing superoxide ion and forming sodium superoxide as the discharge product.

Focusing on the effects of anions^25^ as well as solvent molecules, in this work, we built superoxide stability phase diagrams by using Gutmanns’ DN for solvents and Linert’s DN for anions as double descriptors to confirm the correlation between DNs and superoxide stability. Based on the finding that high DN pairs of anion/solvent encourage the superoxide stability, we demonstrated that durably rechargeable operation of sodium *ion* oxygen cells is possible with the NaSO_3_CF_3_/DMSO.

## Results and discussion

### Superoxide stability prediction by DN

**Figure 1:**
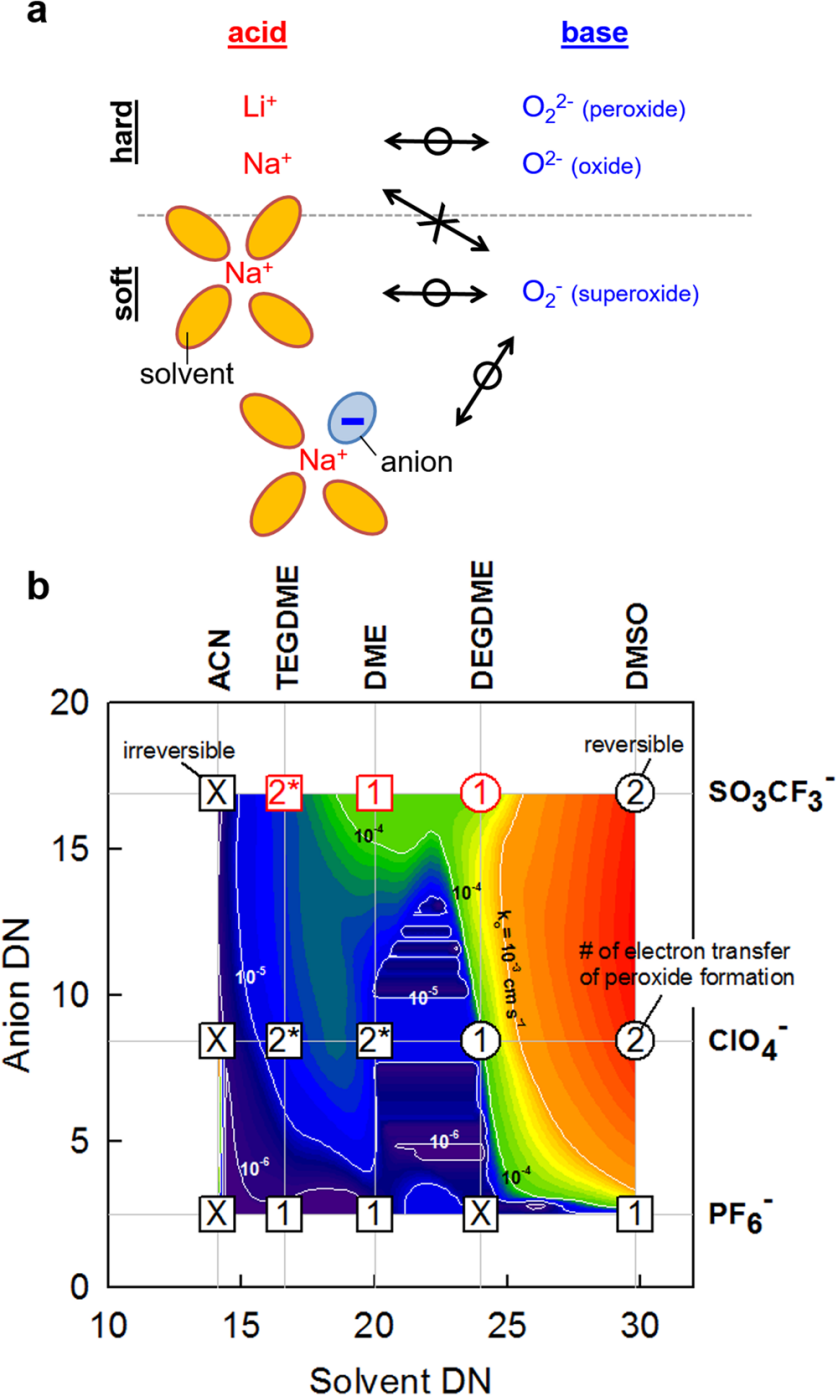
Superoxide stability. (**a**) Interaction between oxygen species (oxide, peroxide or superoxide) and cationic species (bare cations, solvated cations and solvated anion-coordinated cations). The solvation number is not limited to four as shown. Indirect anion coordination where solvent molecules bridge anion and cation is possible even if the direct coordination softens the Lewis acidity of cations more effectively. Bidirectional arrows indicates the interaction between acids and bases: O on the arrows for strong interaction; X for weak or no interaction. (**b**) Contour plots of the standard rate constants (k_o_) of superoxide formation on 2D DN map for superoxide stability. Gutmann’s DN and Linert’s DN values were used for solvents and anions, respectively. The symbols and numbers indicating experimental data points are explained in detail in the text body.

Sodium cation (Na^+^), a hard Lewis acid, is expected to have high affinity for peroxide (O_2_^2−^) and oxide (O^2−^) which are hard Lewis bases^19–21^. Also, the stable ion pair formation between *solvated* Na^+^ and superoxide (O_2_^−^) is expected in solvents of high basicity (high DN value) (Fig. 1a)^19–21^. The Gutmann’s DN values of solvent molecules were reported to be proportional to chemical shifts (δ) of sodium ion after solvation. Therefore, the Gutmann’s DN is understood to describe the environments immediately surrounding sodium ion especially in cases that the contact ion pair formation between the cation and its counter-anions do not perturb the solvation^25^.

In addition to solvents, anions possibly affect the stability of Na^+^-O_2_^−^ formation, which is a lesson from the McCloskey *et al*.’s and Lutz *et al*.’s work showing that stable existence of superoxide was guaranteed by a high DN anion (triflate or CF_3_SO_3_^−^) even with a low-DN solvent (DME)^4,14^. Abraham *et al*. suggested that the coordination of anions to lithium ions in the inner solvation sphere by replacing solvent molecules (from Li^+^(S)_n_ to Li^+^(S)_n-m_(A)_m_ with S = solvent and A = anion) softens the cations more significantly than the simple solvation (solvent coordination) does^26,27^. In a nuclear magnetic resonance (NMR) study from Popov group, strong concentration-dependency of δ of Na was observed with I^−^ and SCN^−^ in various solvents due to coordination of the anions to sodium ion in the inner solvation sphere, in other words, due to the contact ion pair formation between sodium ion and the anions^28^. However, ClO_4_^−^ and tetraphenylborate (BPh_4_^−^) did not show any significant changes in chemical shift with the salt concentrations in the most of solvents used in the study. Considering Linert’s DN values of anions^27,29^, a parallelism is found between the solvation strength of solvent molecules and the coordination strength of anions in terms that the properties are quantified by DN values. That is to say, high-DN anions favor the coordination (similar to solvation): NMR chemical shift of sodium followed the DN order of BPh_4_^−^ (DN = 0) < ClO_4_^−^ (8.44) < I^−^ (28.9) < Br^−^ (33.7)^28^. Also, it explains why low-DN anions such as BPh_4_^−^ or ClO_4_^−^ (not clearly indicated but probably BPh_4_^−^) were used to obtain the perfectly linear relationship between δ and DN values of solvents^25^.

When an ionic salt is dissolved in a solvent, three types of interactions are considered: cation-solvent and anion-solvent interactions based on charge-dipole polarization; and cation-anion electrostatic interaction. From a cation’s standpoint, solvent molecules and counter-anions are competitive in coordination to the cation. Therefore, DN values should be compared between solvents and anions. If one were far higher than the other, only one species would occupy or statistically dominate the inner solvation shell over the other. If they had the DN values within a certain range, both of them could be involved together in the nearest coordination shell. Also, the anion-solvent interaction should be considered in addition to the above discussion. The Linert’s DN values of anions are affected by the acceptor numbers (AN) of solvents even if they were quantified in dichloroethane (DCE; DN = 0, AN = 16.7) as a reference solvent^25,27^. The DN value of an anion decreases when solvent is changed to high-AN one. The *apparent* DN values of anions in extremely high-AN solvents (e.g., water (AN = 54.8), methanol (41.5) and ethanol (37.9)) deviated significantly from the reference value in DCE: e.g. DN of triflate = 16.9 in DCE but -4 in water. However, moderate or low-AN solvents (e.g., DMSO (AN = 19.3) and ACN (18.9)) do not affect the values significantly: e.g. DN of triflate = 16.9, 15.7 and 15.5 in DCE, ACN and DMSO, respectively. Therefore, we could focus on the cation-solvent and cation-anion interactions if the AN values of solvents are less than 30 or more probably 20. Also, the reference values of Linert’s DN were used as a descriptor for coordination to sodium ion or more forward for superoxide stability in the following DN maps since moderate or low-AN solvents have been used for sodium air cells.

In order to consider the interactive effects of solvents and anions on superoxide stability simultaneously, the standard rate constant (k_o_) of oxygen/superoxide electrochemistry was contoured on a two dimensional (2D) DN map (Fig. 1b). The values of k_o_ were used as a criterion measuring superoxide stability, which were obtained from charge transfer resistances measured by the staircase cyclic voltammetry combined with Fourier transform electrochemical impedance spectroscopy (SCV-FTEIS). Empirically, facile electron transfer kinetics of the forward cathodic reaction of the chemically reversible O_2_/O_2_^−^ system resulted in clear existence of anodic peak responsible for its backward reaction. The electrochemical reversibility is interpreted as the superoxide stability because the anodic peaks cannot be found with unstable superoxide generated in the precedent cathodic sweep. According to the 2D DN map, higher DN values of anions as well as solvents stabilized superoxide. Experimental data points (discussed in detail below) were indicated by colored symbols with numbers or X. Circles were used when the anodic peaks responsible for the backward reaction of superoxide formation were observed experimentally. Squares were used for no anodic peak cases, indicating unstable nature of superoxide. Red colors were used for the solvent/anion pairs reported to stabilize superoxide in literatures. The oxygen-to-superoxide reduction was observed irreversible with triflate (SO_3_CF_3_^−^) in DME (0.2 M) and TEGDME (0.5 M) in our cyclic voltammograms even if superoxide was reported to be stable in the same electrolytes in literatures^4,30^. However, the comparatively lower concentration of sodium triflate in our study (0.1 M) than that in these literature works (0.2 M in DME and 0.5 M in TEGDME) is relevant while comparing the superoxide stability in these electrolyte systems. The numbers within the symbols indicate the number of electron transfer for peroxide formation usually following superoxide formation. Peroxide is formed via direct 2e reduction of oxygen or via 1e reduction of superoxide generated at more positive potentials. X indicates no or unclear formation of peroxide. The direct reduction of oxygen to peroxide via 2e transfer is another evidence of superoxide stability.

According to the literatures^4,5,30^ (red symbols in Fig. 1b), all superoxide-stable cases came only from high-DN triflate while other anions resulted in peroxide or oxide formation. Therefore, we could claim a null hypotheses (H_o_) that superoxide stability is solvent-independent or superoxide is always stable with triflate as a high DN-anion or unstable with the other two anions (PF_6_^−^ and ClO_4_^−^) as low DN-anions independent of solvents. To test H_o_, we used dimethyl sulfoxide (DMSO; DN = 29.8) as the solvent, the DN of which is higher than that of DEGDME (DN = 24) that is the highest-DN solvent ever reported for sodium-air cells; and acetonitrile (ACN; DN = 14.1) as the solvent, the DN of which is lower than TEGDME (DN = 16.6) that is the lowest-DN solvent. Three different anions of sodium salts were tested in the solvents for checking superoxide stability: PF_6_^−^ (DN = 2.50) < ClO_4_^−^ (8.44) < CF_3_SO_3_^−^ (16.90) in the increasing order of DN^31^. The coordination strength to Na^+^ is expected to follow the same order.

The superoxide stability results based on electrochemical analysis (discussed below in detail for the DMSO series) rejected the null hypothesis (Fig. 1b). Triflate, the highest-DN anion, failed to form the stable superoxide discharge product in ACN. On the contrary, ClO_4_^−^, which did not form the superoxide complex in previous reports, successfully produced the superoxide in DMSO. Therefore, the Lewis basicities (or coordination to sodium ion) of anions and solvents should be considered simultaneously for expecting superoxide stability. For guaranteeing rechargeable operation of sodium air batteries, anion/solvent pairs should be selected from the kinetically facile region of superoxide formation in the 2D DN map (redder region in Fig. 1b). The required co-consideration of solvents and anions for superoxide stability can be paraphrased to the co-participation of both species as the nearest neighbors in the inner solvation sphere. Higher DN couples resulted in higher superoxide stability leading to higher reversibility in sodium-oxygen cell operation (below in sodium *ion* oxygen cell section): the cells based on CF_3_SO_3_^−^/DMSO were superior to those based on Hartmann’s anion/solvent pair (CF_3_SO_3_^−^/DEGDME)^5^ in terms of reversibility.

### Electrochemical proof on superoxide stability

DMSO is one of the most attractive solvents for lithium-oxygen batteries due to its high polarity, high oxygen solubility and high chemical stability against the intermediates and products of Li-O_2_ electrochemistry, improving rechargeability of Li-O_2_ cells^19,32–35^. High DN value of DMSO would be an additional benefit for steering the discharge mechanism specifically towards superoxide discharge product. However, DMSO has not been studied so far as a solvent for sodium-oxygen batteries because DMSO reacts with sodium metal^36^. Three sodium salts, NaPF_6_, NaClO_4_ and NaSO_3_CF_3_, were compared as supporting electrolytes to study the influence of the anion part of the salt. The results with ClO_4_^−^-based electrolyte were found to be exactly similar to that in CF_3_SO_3_^−^-based electrolyte. Therefore, the results of PF_6_^−^ (DN = 2.5) as the lowest end of DN and CF_3_SO_3_^−^ (DN = 16.9) as the highest end are presented in this report (refer to the supporting information for ClO_4_^−^). Chemical disproportionation or electrochemical conversion of superoxide to peroxide is more possibly discouraged by adopting higher-DN anions.

**Figure 2:**
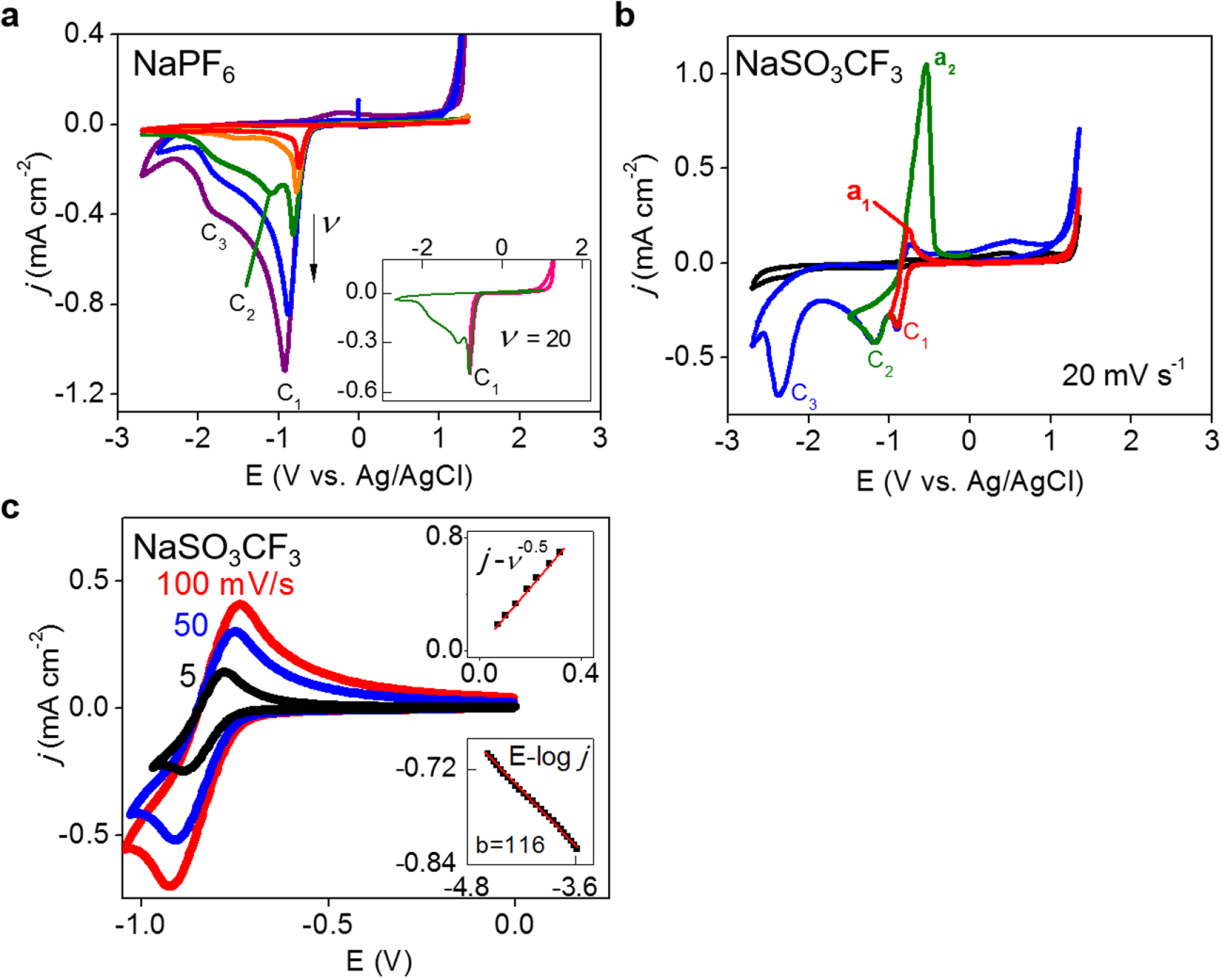
Cyclic voltammograms (CVs; *j* = current density, E = potential). (**a**) Full range CVs recorded at different scan rates in 0.1 M NaPF_6_/DMSO. Scan rates (*υ*, mV s^−1^) increase in direction of the arrow (5, 10, 20, 50 and 100 mV s^−1^). Three sequential cathodic peaks were indicated by c_1_, c_2_ and c_3_. Inset: CVs around peak c_1_ at 20 mV s^−1^ in different potential ranges to confirm the irreversibility. (**b)** CVs recorded at 20 mV s^−1^ at different cathodic potential limits in 0.1 M NaSO_3_CF_3_/DMSO. Anodic peaks were indicated by a_1_ and a_2_ corresponding to the cathodic peaks c_1_ and c_2_ respectively. (**c**) CVs at different scan rates for the first oxygen reduction step in NaSO_3_CF_3_/DMSO. Inset: (top) Scan-rate dependency of cathodic peak current confirming linear relationship between *j* and *υ* ^1/2^; (bottom) Tafel plot (E versus log *j*) for cathodic processes (b = Tafel slope, mV dec^−1^).

The detailed discharge-charge electrochemistry was studied in bulk electrolytes on a glassy carbon electrode by using cyclic voltammograms (CVs). One of the major differences was *irreversibility* of oxygen electrochemistry in PF_6_^−^-based electrolyte versus *reversibility* in CF_3_SO_3_^−^-based electrolyte (Fig. 2; Figure S1 for ClO_4_^−^-based electrolyte). Three cathodic peaks responsible for ORRs (c_1_, c_2_ and c_3_) were identified in both electrolytes (Fig. 2a and b). However, there were no clear anodic peaks observed in PF_6_^−^-based electrolyte while the cathodic peaks were easily matched with the corresponding anodic peaks in the reverse scan for CF_3_SO_3_^−^-based (or ClO_4_^−^-based) electrolytes. Anodic process a_1_ that is the counterpart of the first reduction process c_1_ was not observed with PF_6_^−^ even when potential was scanned to the value just after the c_1_ peak and then reversely scanned (the inset of Fig. 2a). On the contrary, the first ORR step (peak c_1_) with CF_3_SO_3_^−^ were evidently reversible at all scan rates (Figs 2c and S1b).

The scan-rate (*υ*) dependency of cathodic peak currents (*j*_p_) of the first ORR step *for all anions* followed Randles-Sevcik equation (*j*_p_ ~ *υ* ^1/2^), indicating that the electrochemical process is diffusion-limited (top insets of Figs 2c and S1b). From the slope, the diffusion coefficient (*D*_O_) of oxygen in DMSO at 25 °C was calculated to be 1.65 × 10^−5^ cm^2^ s^−1^ in average (with oxygen concentration at 2.1 mM^19^). The cathodic Tafel slopes for the first ORR step in all electrolytes were estimated around 120 mV dec^−1^, indicating an one-electron reduction process (the bottom inset of Figs 2c and S1b). Therefore, the first ORR process at peak c1 is most probably one electron oxygen reduction to superoxide:

1 $${{\rm{O}}}_{2}+{{\rm{Na}}}^{+}+{{\rm{e}}}^{-}={{\rm{NaO}}}_{2}$$

or

2 $${{\rm{O}}}_{2}+{{\rm{Na}}}^{+}{({\rm{S}})}_{{\rm{n}}}+{{\rm{e}}}^{-}={{\rm{Na}}}^{+}{({\rm{S}})}_{{\rm{n}}}{{{\rm{O}}}_{2}}^{-}$$

where Na^+^(S)_n_ = solvated sodium ion. The reversibility between the first ORR (c_1_) and its backward reaction (a_1_) in CF_3_SO_3_^−^-based electrolyte supports the second process (2) indicating stable existence of superoxide ion possibly in a form of an ion pair with solvated sodium cation *in electrolyte* ^19^. On the other hand, superoxide instability in the presence of PF_6_^−^ is confirmed by the c_1_/a_1_ irreversibility. Any indications of reversibility in CVs were not observed even at higher concentrations (0.3 M NaPF_6_ in DMSO in Figure S3) and at higher scan rates even if higher cation concentrations more possibly stabilize superoxide and peroxide and a higher scan rate of 130 mV s^−1^ was used to detect the unstable superoxide-containing complex^20^. The anion-dependent reversibility of superoxide formation appears to be clear and should be emphasized because the acid-base interactions between cations and solvent molecules have been dominantly considered until now.

**Figure 3:**
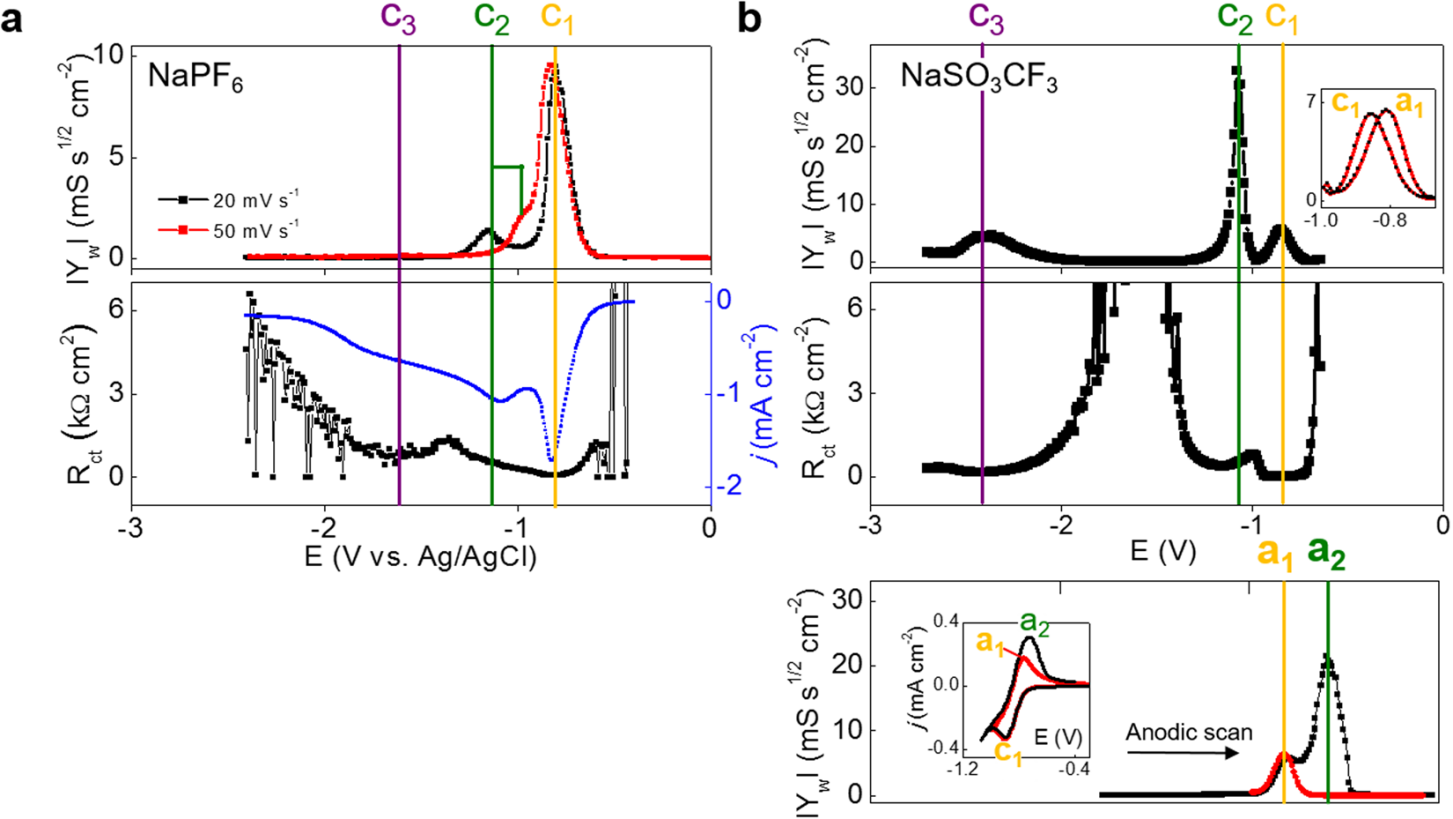
Real-time impedance analysis during cathodic or anodic potential scans. (**a**) and (**b**) Warburg admittance (*Y*_w_) and charge transfer resistance (R_ct_) in 0.1 M NaPF_6_/DMSO and NaSO_3_CF_3_/DMSO in a cathodic scan (*υ* = 20 mV s^−1^). Bottom panel of b: Warburg admittance obtained during anodic potential scan following cathodic scan from 0 V to either −0.99 or −1.8 V as the cathodic potential limit. Inset: (Top in **b**) Warburg admittance peaks in both cathodic and anodic scans. (Bottom in **b**) CVs in the potential ranges around superoxide/oxygen electrochemistry. Red and black curves were obtained by scanning cathodically to −0.99 V and −1.07 V respectively and then scanning anodically. The anodic peak current (a_1_) significantly increased when the previous cathodic scan was extended to the more negative potential involving peroxide formation reaction (peak a_2_).

A second cathodic peak (peak c_2_ in Figs 2 and S1) was clearly observed with CF_3_SO_3_^−^ (or ClO_4_^−^) at all scan rates. However, it was observed only at 20 mV s^−1^ in the PF_6_^−^-based electrolyte. The peak c_2_ at higher scan rates appears to be buried in the first reduction wave around peak c_1_ of high charging currents^37^. The lack of peak c_2_ at slower scan rates (<20 mV s^−1^) in the PF_6_^−^-based electrolyte indicates that the products formed from the previous step at peak c_1_ are unstable. To clearly reveal the presence of the second cathodic process (c_2_) in PF_6_^–^-containing DMSO at higher scan rates, we used the combined SCV-FTEIS technique by which the transient natures of electrochemical reactions can be kinetically snapshotted while the electrochemistry with potential variation is investigated in real time^38–40^ (Figure S4; Experimental details in the supporting information). A large body of impedance spectra obtained along cathodic scan was analyzed by a two-RC equivalent circuit (Figure S4b)^41^. Two successive diffusion-controlled electron transfer processes were identified at 20 mV s^−1^ by two peaks (c_1_ and c_2_) in Warburg admittance (*Y*_w_) profile of PF_6_^−^-based electrolyte (the top panel of Fig. 3a). The second *Y*_*w*_ peak (c_2_) was partially overlapped with the c_1_ peak but identifiable at 50 mV s^−1^ that is the scan rate not showing the clear c_2_ peak in CV (Fig. 2a) due to high charging current. The reaction proceeding at peak c_1_ was confirmed as an electrochemically quasi-reversible one-electron reduction because its half-width value (Δ*E*_1/2_) was ~115 mV larger than the ideal value for completely reversible systems (=90.6/n mV with number of electron transfer, n = 1)^39,40^. The even larger value of Δ*E*_1/2_ at 155 mV for the second Warburg peak (c_2_ observed at 20 mV s^−1^) supports that the second ORR step is one-electron reduction of superoxide to peroxide which has more shifts towards the irreversible extreme of electrochemical reduction:

3 $${{{\rm{O}}}_{2}}^{-}+{\rm{2}}\,{{\rm{Na}}}^{+}+{{\rm{e}}}^{-}={{\rm{Na}}}_{2}{{\rm{O}}}_{2}.$$

The cathodic process at c_3_ with PF_6_^−^ is possibly assigned to the reduction of surface-adsorbed peroxide to oxide (Na_2_O) because its charge transfer resistance (*R*_ct_) was clearly minimized at its potential (the bottom panel of Fig. 3a) without any Warburg peak (the top panel of Fig. 3a). However, the oxide formation occurring only at high overpotentials is of little importance in Na-O_2_ battery electrochemistry^12^.

From the viewpoint of *electrochemical* reversibility of superoxide, the *chemical* stability of superoxide generated at c_1_ should be discussed in low DN situations, which is more important than assigning voltammetric peaks c_1_, c_2_ and c_3_ to the corresponding *electrochemical* processes. We could reason the *chemical* reactions causing the instability from experimental clues even if it is difficult to identify them obviously. Disproportionation of superoxide to peroxide was reported by previous works^18,20^, which is thought to be one of the most possible chemical processes responsible for the superoxide instability in low DN situations:

4 $${{\rm{2NaO}}}_{2}\ast ={{\rm{Na}}}_{2}{{\rm{O}}}_{2}\ast +{{\rm{O}}}_{2}$$

where * indicates surface species. The absence of peak c_2_ for superoxide reduction (equation 3) at slow scan rates (<20 mV s^−1^) is due to the superoxide-to-peroxide conversion (equation 4) leaving no reactants for equation 3. However, the c_2_ peaks were identified by SCV-FTEIS at fast scan rates because there were no time enough for disproportionation to proceed. Therefore, the disproportionation reaction is considered quasi-reversible or not very fast. On the other hand, no peroxide oxidation peak a_2_ observed at slow scan rates in PF_6_^−^-containing electrolyte (Fig. 2a) is against the existence of peroxide generated by disproportionation. The experimental fact would not be contradictory to disproportionation if peroxide were also unstable in the presence of PF_6_^−^. Peaks a_2_ for peroxide oxidation found at fast scans (>100 mV s^−1^; Figs 2a and S2) supports the chemical instability of peroxide in a not very fast kinetics. In summary on electrochemistry in low DN electrolytes, oxygen is reduced with one electron to quasi-stable superoxide that is chemically disproportionated to quasi-stable peroxide.

Since voltammetric results indicated stable superoxide in CF_3_SO_3_^−^ (or ClO_4_^−^)-based electrolyte, adsorption of ORR products or intermediates on electrode surface is possibly neglected in impedance interpretation by removing film-related parameters (R_f_ and C_f_ in the equivalent circuit of Figures S4b, S5). By Δ*E*_1/2_ of Warburg peaks at 112 mV, both of the first cathodic process (c_1_; oxygen to superoxide) and its backward reaction (a_1_) were confirmed quasi-reversible one-electron processes (the inset of the upper panel in Fig. 3b; Figure S6 for ClO_4_^−^). The standard rate constant (*k*°) for the ORR to superoxide were calculated to be 7.40 × 10^−3^ (or 7.05 × 10^−3^) cm s^−1^ in CF_3_SO_3_^−^ (or ClO_4_^−^)-based electrolytes by using their *R*_ct_ minimum value in *i*_0_ = *RT/nFR*_ct_ and *k* ^0^ = *i*_0_/*nF* ${C}_{O}^{\ast }$ (the middle panel in Figs 3b and S5). The *k* ^*o*^ values are more than one order higher than that of superoxide formation in Li^+^-containing DMSO (*k* ^0^ = 0.21 × 10^−3^ cm s^−1^), being in the same order of magnitude with that of tetrabutylammonium (TBA^+^)-containing DMSO (*k* ^0^ = 17 × 10^−3^ cm s^−1^)^19^.

Importantly, the second ORR step (c_2_) with CF_3_SO_3_^−^ (or ClO_4_^−^) was estimated to be a *two-electron* reduction of molecular oxygen to peroxide:

5 $${{\rm{O}}}_{2}+2\,{{\rm{Na}}}^{+}+{{\rm{2e}}}^{-}={{\rm{Na}}}_{2}{{\rm{O}}}_{2}.$$

ΔE_1/2_ value at ~51 mV of the Warburg peak (Figs 3b and S5) indicated the two electrons (≈90.6/*n* mV with *n* = 2) while the n value was estimated at 1.9 from scan-rate dependency of peak potential and current (Nicholson and Shain relationship; Figure S7). It means that the superoxide formed in the first ORR step is too stable in these electrolytes to be reduced at the potential around peak c_2_. The anodic peak a_1_, which was not clearly identified in CV due to peak a_2_ in higher intensity (green in Figs 2B and S1a), was clearly identified in Warburg impedance profile obtained during anodic scan following cathodic scan from 0 V to either −0.99 or −1.8 V as the cathodic potential limit (the bottom panel of Figs 3b and S5). The survival of superoxide even after experiencing potentials more negative to peroxide formation confirms the stability of superoxide. Instead of superoxide, however, oxidation of other electrolyte constituents is possibly suspicious in CF_3_SO_3_^−^ (or ClO_4_^−^)-based electrolyte during anodic scans, when considering that the charge under the oxidation peak a_2_ (Figure S7) is considerably higher than that under the reduction peaks c_1_ and c_2_. The electrolyte constituents could be solvent, salt or their decomposition products formed in the presence of ORR products/intermediates^42–45^. However, the possibility is limited to the situation that potential is cathodically scanned to the peroxide formation potential. No additional charges were developed during anodic scans when the cathodic scan was restricted to the potential of superoxide formation up to −0.99 V (the inset of the bottom panel of Figs 3b and S5).

The peak c_3_ with CF_3_SO_3_^−^ (or ClO_4_^−^) in DMSO (Figs 2 and S5) could be assigned to superoxide reduction when considering that the superoxide reduction was reported at the same potential (~−2.5 V) in TBAPF_6_ or KPF_6_ in ACN^20^. The disappearance of peak a_2_ after experiencing peak c_3_ in the previous cathodic scan indicates that the species oxidizable at peak a_2_ as well as superoxide are reduced at peak c_3_. However, the reactions occurring at such highly cathodic overpotentials (peak c_3_) are of little importance in sodium-air battery electrochemistry.

### Sodium *ion* oxygen batteries based on high-DN anion/solvent pairs

The voltammetric and SCV-FTEIS studies in NaSO_3_CF_3_ as well as NaClO_4_ based DMSO clearly show the stability of superoxide in solution phase. However, the superoxide in NaPF_6_ based electrolyte undergoes immediate disproportionation to the surface adsorbed Na_2_O_2_. The potential of the Na-O_2_ cell remains relatively unaltered at the ORR to superoxide step because oxygen is supplied continuously. Therefore, NaO_2_ would precipitate as stable discharge product in NaSO_3_CF_3_ as well as NaClO_4_ based Na-O_2_ cells whereas Na_2_O_2_ would be the discharge product in NaPF_6_ based Na-O_2_ cell. However, the Na_2_O_2_ cannot have a stable existence in electrolyte due to its fast chemical reaction with electrolyte resulting in decomposition products as evident from voltammetric results.

**Figure 4:**
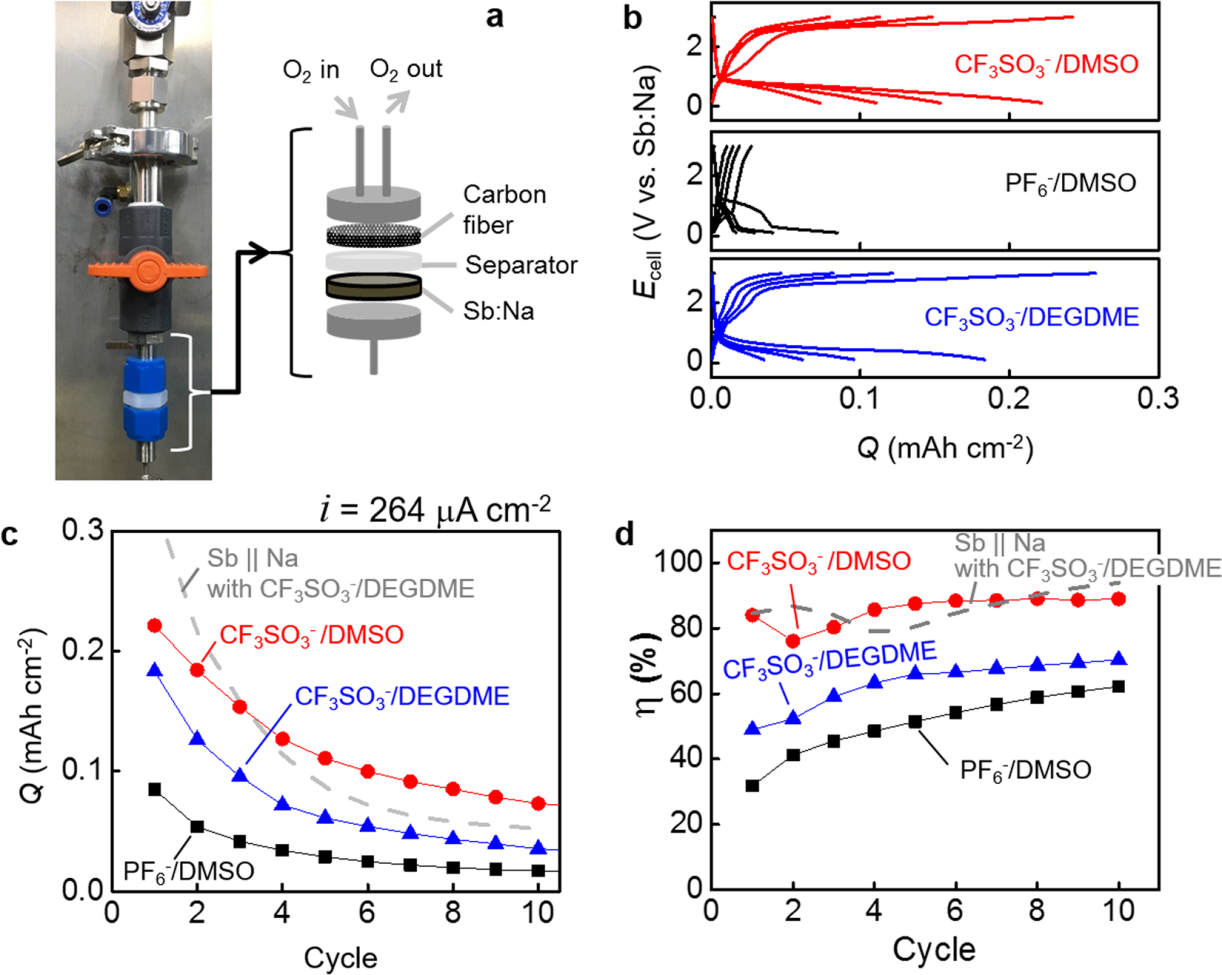
Sodium *ion* oxygen (Na^+^/O2) batteries of Sb:Na||O_2_. (**a**) The cell consisting of a pre-sodiated antimony (Sb:Na) anode and a carbon fiber paper cathode with a glass fiber separator. Geometric electrode area = 1.23 cm^2^. (**b**) Potential profiles of charge and discharge at 264 μA cm^−2^ at the 1^st^, 3^rd^, 5^th^ and 10^th^ cycles. The cycle numbers were counted after the initial discharge. (**c**) Capacity (Q) retention along cycles. (**d**) Coulombic efficiencies (η) along cycles. The data of CF_3_SO_3_^−^/DEGDME were shown up to the third cycle. After the third cycle, the values were severely fluctuated because the values of capacity were very small. The small measurement errors in capacity are possibly amplified in η.

The superoxide-stabilizing benefit of the high DN pair (CF_3_SO_3_^−^/DMSO) was realized in sodium *ion* oxygen (Na^+^/O_2_) batteries (not conventional sodium metal oxygen (Na^0^/O_2_) batteries). Pre-sodiated antimony (Sb:Na) was used as an anode during discharge instead of sodium metal because DMSO is reacted with the metal. The Sb:Na was prepared electrochemically by sodiating Sb nanoparticles in a cell of Sb||Na metal with 1 M NaClO_4_ in a mixture of ethylene carbonate and propylene carbonate (EC:PC)^46^–^48^. The pre-sodiation of Sb and its backward reaction was highly reversible in the electrolyte with the capacity (*Q*_dNa_) at 1.1 mAh cm^−2^ or 550 mAh g_Sb_^−1^ (Figure S8). In the Na^+^/O_2_ cells of Sb:Na||O_2_, the high DN pair (CF_3_SO_3_^−^/DMSO) showed higher reversibility than the Hartmann’s anion/solvent pair (CF_3_SO_3_^−^/DEGDME)^5^ and its low-DN anion control (PF_6_^−^/DMSO) (Fig. 4). 33% of the discharge capacity at the first cycle after the initial discharge (the 0^th^ cycles) was obtained after 10 cycles with 86% as the coulombic efficiency averaged over ten cycles. The reversibility of the CF_3_SO_3_^−^/DMSO is recognized as a stark improvement when considering only negligible capacities were obtained after two cycles in its counterparts. Even in the most improved cells in literatures, it was difficult to find the sodium oxygen cells delivering capacities over 10 cycles of *full* charge and discharge^5,46^. In the most of works exhibiting durable long-cycle operation of sodium oxygen batteries, the cells were operated only at the low depth of discharge or discharged up to a limited capacity cut-off to prevent the formation of large amount of ORR products from being formed^8,30^. The superoxide-stabilizing benefit of the high DN pair (CF_3_SO_3_^−^/DMSO) in the sodium *ion* oxygen (Na^+^/O_2_) batteries was confirmed by identifying products. Sodium superoxide was identified in X-ray diffraction pattern, which was the product formed after full discharge of the CF_3_SO_3_^−^/DMSO-based cells (Figure S9). On the other hand, NaOH was the discharge product in PF_6_^−^/DMSO-based cells, which unstable peroxide is converted to in low DN environments.

The comparison of the Sb:Na||O_2_ with Sb||Na clarifies the superiority of the high DN pair (CF_3_SO_3_^−^/DMSO) to its counterparts. The Sb showed serious capacity decay in the cell of Sb||Na with CF_3_SO_3_^−^/DEGDME while the sodiation/de-sodiation of Sb was highly reversible in ClO_4_^−^/EC:PC (sodium ion battery half cells) (Figure S8b). The inefficient sodiation of Sb is responsible for the decay probably due to the strong solvation of Na^+^ by DEGDME: the sodiation capacity in the following cycle (Q_Na,i_) was significantly less than the desodiation capacity at the previous cycle (Q_dNa,i-1_). However, the coulombic efficiency (η) at each cycle is relatively high (Q_dNa,i-1_> Q_Na,i_~Q_dNa,i_). The sodiation/desodiation in Sb||Na cells is kinetically limited to the electrochemical processes of Sb because the deposition/dissolution of Na metal is kinetically fast and highly reversible. When the Na metal was replaced by the air cathode (carbon fiber paper) with oxygen supply in CF_3_SO_3_^−^/DEGDME, the capacity decay was more serious (grey dashed lines for Sb||Na versus blue triangles for Sb:Na||O_2_ in Fig. 4c and d). The poor reversibility on the air electrode (poorer than the reversibility between metal deposition and dissolution in Sb||Na) limited the cyclability performance. On the contrary, the high DN pair CF_3_SO_3_^−^/DMSO guaranteed better cyclability (red circles in Fig. 4), indicating the oxygen electrochemistry is reversible at least as comparable as Na^0^/Na^+^ electrochemistry as the metal-side reaction of Sb||Na battery cells.

Even if the DMSO was suggested as the highest DN solvent in this work, it had the serious problem of the reactivity with sodium metal. Therefore, we demonstrated its good reversibility of oxygen electrochemistry by constructing the novel device sodium *ion* oxygen batteries instead of the conventional sodium metal oxygen batteries. In turn, unfortunately, the Na/Sb alloying reaction in the high DN solvent (DEGDME and probably DMSO even if the DMSO cannot be tested in Sb||Na cells) were not as reversible as in the conventional electrolyte EC + PC for rechargeable batteries. The high DN was favored for the superoxide stability in oxygen electrochemistry but not favored for Na/Sb alloying reaction. Therefore, we are looking for the high DN solvent that is not reactive with sodium metal for sodium *metal* oxygen cells. Alternatively, novel anode materials guaranteeing high reversibility even in high DN solvent should be found for the novel sodium *ion* oxygen cells.

## Conclusions

High-DN pairs of anion/solvent (e.g., CF_3_SO_3_^−^/DMSO) was proposed as an electrolyte for reversible operation of Na-O_2_ battery since they were expected to stabilize the superoxide. The electrolyte-dependency of the Na-O_2_ electrochemistry was investigated from mechanism and reversibility viewpoints by SCV-FTEIS. This study showed for the first time in literature that the nature of discharge products (NaO_2_ or Na_2_O_2_), battery performances and the discharge-charge reversibility greatly depend on the anion part of the sodium salt. The superoxide formed as the first step ORR product in CF_3_SO_3_^−^/DMSO and ClO_4_^−^/DMSO was very stable so that the formation of Na_2_O_2_ occurred only by a second ORR step of electrochemical two-electron reduction of molecular oxygen to peroxide (not by one-electron reduction of superoxide). In PF_6_^−^/DMSO, however, the superoxide immediately disproportionated to the surface adsorbed peroxide. Therefore, the discharge product in Na-O_2_ cells with CF_3_SO_3_^−^ or ClO_4_^−^/DMSO was superoxide (NaO_2_), whereas peroxide (Na_2_O_2_) was identified as the discharge product in PF_6_^−^/DMSO. The reversibility and the battery performances of CF_3_SO_3_^−^/DMSO-based superoxide cells were promising. This study clearly reveals the importance of a proper selection of anion/solvent pairs of electrolyte for achieving the good reversibility and performance for Na-O_2_ cells by controlling the nature of discharge product.

## Methods

### Electrochemical characterization

All chemical were stored and manipulated in a glove box or a dry room. An air-tight single compartment glass cell was used for cyclic voltammetry (CV) and combined staircase cyclic voltammetry-Fourier transform electrochemical impedance spectroscopy (SCV-FTEIS). High-purity dry O_2_ or N_2_ was introduced into the cell during the experiments. Glassy carbon (GC) disk electrodes were used as a working electrode with home-made Ag/AgCl reference electrodes. 0.1 M solutions of different sodium salts in aprotic solvents were used for the electrochemical characterization: anion of salt = hexafluorophosphate (PF_6_^−^), perchlorate (ClO_4_^−^) or triflate (CF_3_SO_3_^−^); solvent = acetonitrile (ACN), dimethoxy ethane (DME), diethylene glycol dimethyl ether (DEGDME) or dimethyl sulfoxide (DMSO). The SCV-FTEIS (staircase cyclic voltammograms with Fourier transform electrochemical impedance spectroscopy) experiments were carried out with a homemade fast-rise potentiostat, a Hewlett-Packard HP 33120 A arbitrary waveform generator and a National Instrument NI-5922 high speed data acquisition system controlled by a computer.

### Sodium *ion* oxygen (Na^+^/O_2_) cells

Sb nanoparticles were synthesized by reducing Sb^3+^ in ethanol by NaBH_4_ for 3 h (0.6 g NaBH_4_ in 50 mL ethanol + 1 g SbCl3 in 50 mL ethanol)^45^. They were pre-sodiated in an electrochemical cell of Sb||Na. 1 M NaClO_4_ in EC/PC was used, where EC = ethylene carbonate and PC = propylene carbonate. The Sb electrodes of Sb||Na were prepared by coating an aqueous slurry mixture of Sb, carbon black (Super-P) and a binder in a 70:15:15 wt. ratio on a copper foil. PAA (polyacrylic acid)/pullulan was used as the binder. Loading density of Sb was 2.4 mg_Sb_ cm^−2^. The 2032 coin cells were used for the Sb||Na. The capacity of the pre-sodiated Sb electrode (Sb:Na) was measured at 550 mAh g^−1^. Oxygen cells were constructed by using the Sb:Na as the anode, carbon fiber paper (AvCarb P50) as the cathode and glass fiber separator (Whatman GF/D). Oxygen pressure was controlled at 770 torr by a throttle valve.

## Electronic supplementary material

supporting information
