# Supplementary material for: Superoxide stability for reversible Na-O2 electrochemistry
Source: Sci Rep. 2017 Dec 15;7:17635. doi: 10.1038/s41598-017-17745-9 (PMC5732307; doi:10.1038/s41598-017-17745-9)
Supplement: Supplementary file 1 — supporting information [file 41598_2017_17745_MOESM1_ESM.doc]

Supporting Information

Superoxide stability for reversible Na-O_2_ electrochemistry

V. S. Dilimon,^1, †^ Chihyun Hwang,^1, †^ Yoon-Gyo Cho,^1^ Juchan Yang,^1^ Hee-Dae Lim,^2^ Kisuk Kang,^2^ Seok Ju Kang,^1^ and Hyun-Kon Song^1, *^

^1^School of Energy and Chemical Engineering, UNIST, Ulsan 44919, Korea

^2^Department of Materials Science and Engineering, Research Institute of Advanced Materials (RIAM), Seoul National University, Seoul 08826, Korea

^†^These authors contributed equally to this work

Corresponding Author

philiphobi@hotmail.com (H.-K.S.)

***Experimental details***

**Chemicals.** The following chemicals were used as received immediately after received (from Sigma-Aldrich) or stored in a glove box: anhydrous dimethyl sulfoxide (DMSO, >99.9 %), anhydrous diethylene glycol dimethyl ether (DEGDME, >99.5 %), propylene carbonate (PC, >99.7%) sodium hexafluorophosphate (NaPF_6_, >98 %), sodium triflate (NaSO_3_CF_3_, >98 %), sodium perchlorate (NaClO_4_, >98 %), antimony chloride (SbCl3, >99.95 %), sodium borohydride (NaBH4 >99 %) and sodium metal.

**Sb Synthesis.** Sb nanoparticles as an anode material were synthesized by chemical reduction of SbCl_3_ with NaBH_4_. NaBH_4_ (1.2 g) and SbCl_3_ (2.0 g) were mixed in ethanol (200 ml) and stirred for 3 h at room temperature. Nanoparticles were harvested from the mixture by filtration, washed and dried under vacuum at 80 ^o^C.

**Cells.** A single compartment glass cell with an air-tight Teflon lid having provision to insert electrodes air-tightly was used for the cyclic voltammetry (CV), and combined staircase cyclic voltammetry-Fourier transform electrochemical impedance spectroscopy (SCV-FTEIS). High-purity dry O_2_ or N_2_ was introduced into the cell through a Teflon tube while it also flowed out from the cell through another Teflon tube. Joints were sealed with Teflon tape and parafilm. The cell was assembled in a dry room. Glassy carbon (GC) working electrode with 0.2826 cm^2^ geometric area was polished sequentially with 1 and 0.3 μm alumina slurry and then sonicated for 1 minute in ultrapure water (18.2 MΩ cm). The electrode was then washed with ultrapure water, and dried thoroughly with a stream of high purity Ar. A platinum mesh was the counter electrode. An Ag/AgCl wire inserted into a glass tube was used as the reference electrode. AgCl was made on a flame cleaned Ag wire in 0.1 M HCl by cycling between 0 to 0.5 V at 20 mV s^-1^ for 3 cycles. Reference electrode was prepared daily for each set of experiments and its potential stability was often checked versus an Ag/Ag^+^ (0.01 M) in acetonitrile since the electrochemical experiments were in aprotic solvents.

**Voltammetry and Impedance spectroscopy.** Voltammetric experiments were performed with an IVIUMSTAT electrochemical interface. SCV-FTEIS experiments were carried out with a homemade fast-rise potentiostat, a Hewlett-Packard HP 33120A arbitrary waveform generator and a National Instrument NI-5922 high speed data acquisition system controlled by a computer. A staircase potential program with potential steps of 10 mV height was applied to the GC working electrode from one potential to another in the cathodic direction and then in the reverse anodic direction to the starting potential. The potential was held constant for 200 ms after each potential step. The SCV scan rate was 20 mV s^-1^. Sampling rate for both stepped potential and the resulting current was 50 kHz. After the data acquisition, the data from both the voltage step and the resulting chronoamperometric current were segmented for each potential step. The current for the last 10 ms for each potential step was averaged and the resulting SCV was recorded. Impedance data were then computed by taking the first derivatives of the stepped voltage and the resulting current with respect to time, then by fast Fourier transform of the derivative voltages and current signals into ac voltages and currents in the frequency ranging from 1/*t*_total_ as the low frequency limit to 1/(2Δ*t*) as the high frequency limit. Where, Δ*t* = 1/(sampling rate). Matlab program (Maths Works, Natick, MA) was used for this process.

**Reversibility measure by Warburg admittance.** ORR Reversibility was estimated by the half-width value (Δ*E*_1/2_) around the half-wave potential (*E*_1/2_) of a Warburg admittance (*Y*_w_ = 1/*Z*_w_) peak: a symmetric peak at *E_p_* (peak potential) = *E*_1/2_ with Δ*E*_1/2_ = ±1.76 (*RT/nF*) = 90.6/*n* mV for ideally reversible processes.^S1, S2^ However, the shape, *E_p_* and Δ*E*_1/2_ of Warburg peaks for irreversible and quasi-reversible reactions depend on the number of electrons transferred (*n*), exchange rate constant (*k*^0^), diffusion coefficient (*D*), transfer coefficient (*α*) and step period (*t*_p_).

***n* calculation.** In order to understand the second ORR step in NaSO_3_CF_3_ and NaClO_4_ based electrolytes in further detail, we examined the reaction as a function of sweep rate (Figure S6). The peak potential shifted to more negative values with scan rate. The peak current (*i*_p_) varies linearly with the square root of sweep rate (*υ*^1/2^) and the straight line passes through the origin as per the Nicholson and Shain relationship:


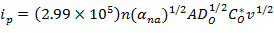
 (S1)

where A is the electrode area,
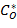
 is the oxygen concentration (2.1 mM)^S3^, and all other parameters have their usual meaning. The
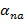
 values for the reaction in NaSO_3_CF_3_ and NaClO_4_-based electrolytes are determined to be 0.295 and 0.251 respectively from the plot of peak potential (*E*_p_) versus log *υ* (the inset of Figure S6) by using the relation:


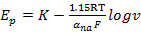
 (S2)

The number of electrons transferred (*n*) in the second ORR step (peak c_2_) was calculated to be 1.9 by using these
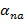
 values in Eq. S1. Also, *D*_o_ was calculated by Randles-Sevcik equation (Eq. S3):


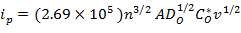
 (S3).

This observation confirms the SCV-FTEIS results that a two electron reduction of oxygen to peroxide is the second ORR step in NaSO_3_CF_3_ and NaClO_4_ based electrolytes.

**Sodium ion air battery (Na^+^/O_2_) cells.** Sb electrodes were sodiated in a standard CR2032 coin cell of Sb composite electrode | separator (Whatman, GF/D) | Na metal. The Sb composite electrodes were a mixture of Sb nanoparticles, carbon black (Super P) and a composite binder (polyacrylic acid/pullulan) in a weight ratio of 70:15:15. The Sb electrodes were sodiated up to 0.01 V_Na/Na+_ and de-sodiated up to 2.5 V_Na/Na+_ galvanostatically at 33 mA g^-1^ for the solid-electrolyte interphase (SEI) layer formation. Then, 10 cycles of sodiation/de-sodiation at 66 mA g^-1^ followed for stabilizing the Sb electrodes. Finally, they were galvanostatically sodiated up to 0.01 V_Na/Na+_ at 33 mA g^-1^. The pre-sodiated Sb electrodes (Sb:Na) were used in the following Na^+^/O_2_ cells by taking the compartment after disassembling the coin cells. A Swagelok-type cell configuration was used for making the Na^+^/O_2_ cells. An air cathode (AvCarb P50) and the Sb:Na anode were assembled with a separator (Whatman, GF/D) in an argon atmosphere glove box. The assembled Na^+^/O_2_ cells (Sb:Na || O_2_) were electrochemically characterized under oxygen atmosphere at 770 Torr. The pressure was maintained by using a throttle valve.


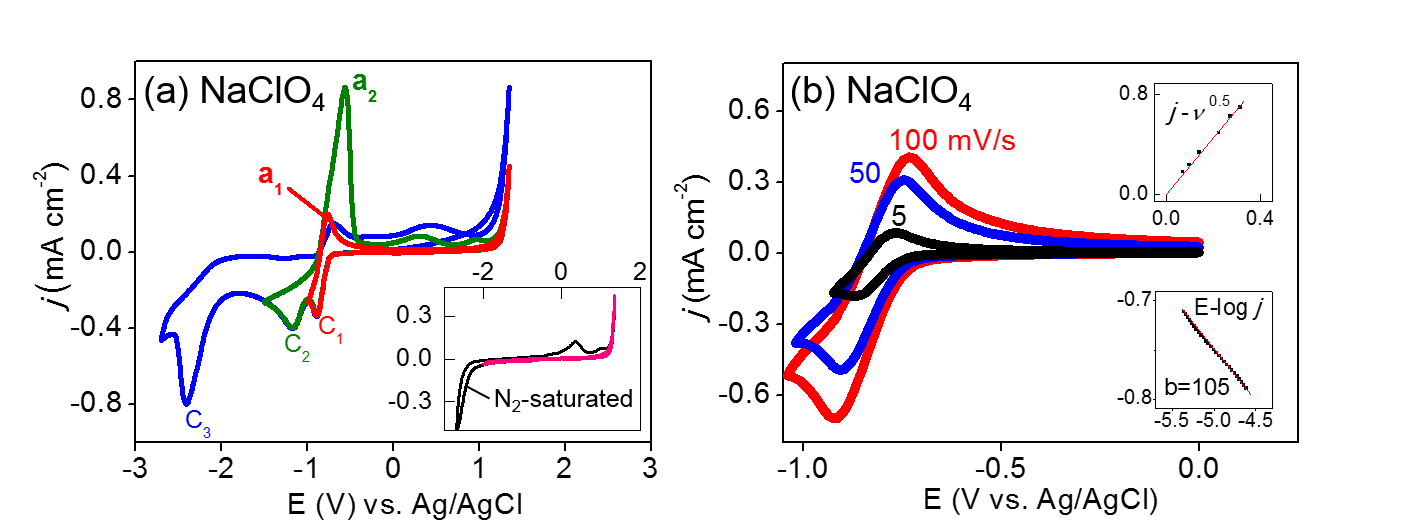


**Figure S1 | Cyclic voltammograms** **of 0.1 M NaClO_4_/DMSO** (CVs; *j* = current density, E = potential)**.** (**a**) CVs recorded at 20 mV s^-1^ at different cathodic potential limits. Cathodic and anodic peaks were indicated by c and a followed by subscripted number, respectively. Inset: The anodic peak at ~0.5 V, observed in N_2_-saturated electrolyte, was absent in O_2_-saturated electrolyte. (**b**) CVs at different scan rates for the first oxygen reduction step. Inset: (top) Scan-rate dependency of cathodic peak current confirming linear relationship between *j* and *υ*^½^; (bottom) Tafel plot (E versus log *j*) for cathodic processes (b = Tafel slope, mV dec^-1^).

**Figure S2 | Oxidation peak for Na_2_O_2_ at high scan rates.** The CVs in 0.1 M NaPF_6_ / DMSO.


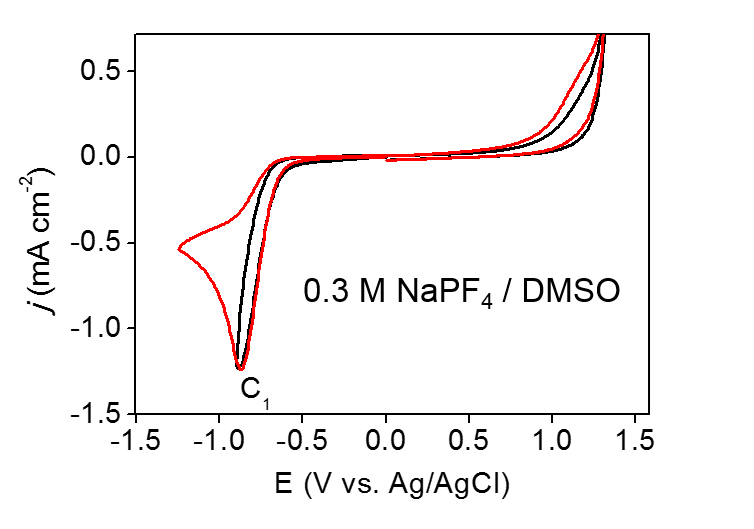


**Figure S3 | ORR irreversibility at peak c_1_.** The CVs at 130 mV s^-1^ in 0.3 M NaPF_6_ / DMSO.


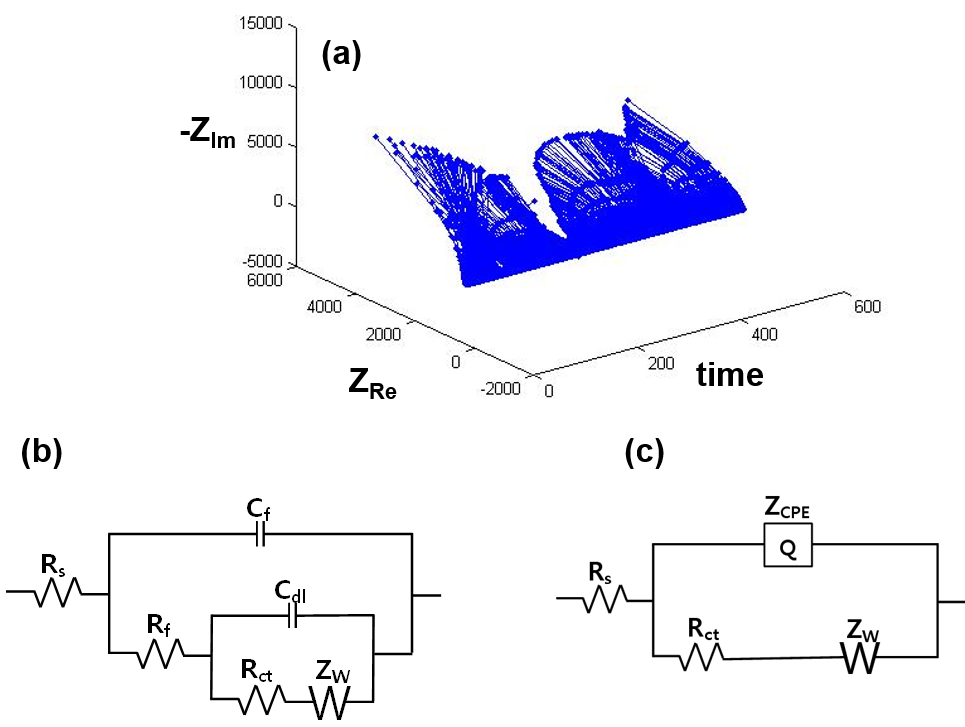


**Figure S4 | Combined SCV-FTEIS.** (**a**) A typical SCV-FTEIS data. A large body of impedance data was recorded during potential sweep in every 10 mV intervals. (**b** and **c**) The equivalent circuits used to analyze PF_6_^-^-based and CF_3_SO_3_^-^-based electrolytes, respectively. *R*_s_ = solution resistance; *R*_f_ and *C*_f_ = resistance and capacitance components due to the surface adsorbed discharge products/intermediates, respectively; *C*_dl_ = the double layer capacitance; *R*_ct_ = the charge transfer resistance; *Z*_w_ = Warburg impedance.


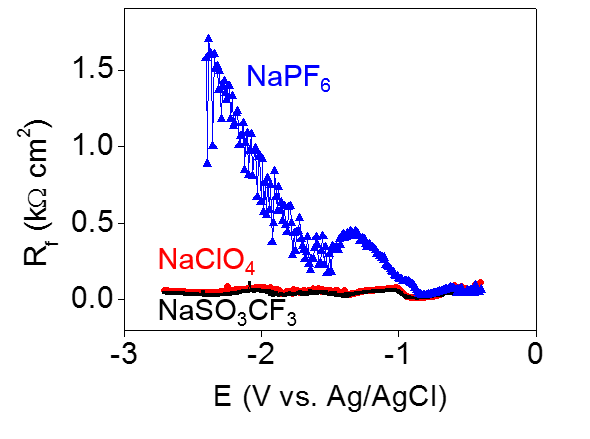


**Figure S5 |** **Profiles of the resistances** caused by the surface-adsorbed species (R_f_) along cathodic potential scan in oxygen-saturated DMSO electrolytes.

Since voltammetric results indicated the stable superoxide in CF_3_SO_3_- (or ClO_4_-)-based electrolyte, adsorption of ORR products or intermediates on electrode surface is possibly neglected in impedance interpretation by removing film-related parameters (R_f_ and C_f_ in the equivalent circuit of Figure S3b). There were no significant difference in goodness of fit between the simple Randles circuit (Figure S3c) and the two-parallel-RC-containing circuit used for NaPF_6_-based electrolyte (Figure S3b). Also, the insignificant change of small values of R_f_ over the whole range of potential was observed in NaSO_3_CF_3_-based (or NaClO_4_-based) electrolyte, when compared with NaPF_6_-based electrolyte (Figure S4). The surface film resistance showed significant increase during the cathodic scan in NaPF_6_-based electrolyte. The serious ORR product adsorption supports the results of Liu *et al*. that more serious polarization was developed in the presence of NaPF_6_ rather than NaClO_4_ in DME.^S4^


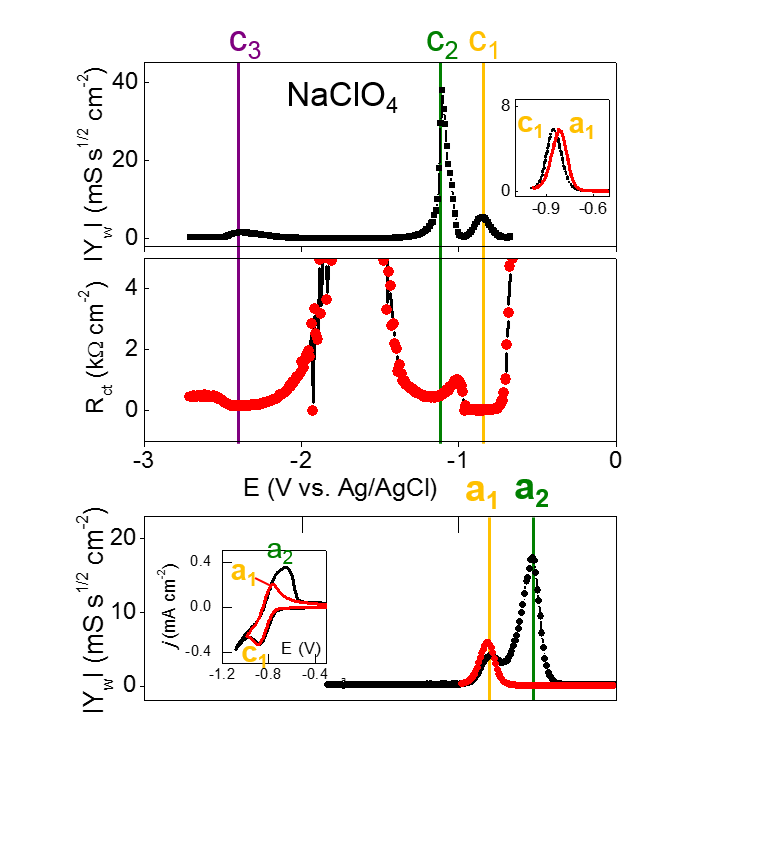


**Figure S6 | Real-time impedance analysis of 0.1 M NaClO_4_/DMSO during cathodic or anodic potential scans.** Warburg admittance (*Y*_w_) and charge transfer resistance (R_ct_) in a cathodic scan (*υ* = 20 mV s^-1^; except of the bottom panel). Bottom panel of **b**: Warburg admittance obtained during anodic potential scan following cathodic scan from 0 V to either -0.99 or -1.8 V as the cathodic potential limit. Inset: (Top) Warburg admittance peaks in both cathodic and anodic scans. (Bottom) CVs in the potential ranges around superoxide/oxygen electrochemistry. The anodic peak current (a_1_) significantly increased when the previous cathodic scan was extended to the potentials for peroxide formation reaction (peak a_2_).


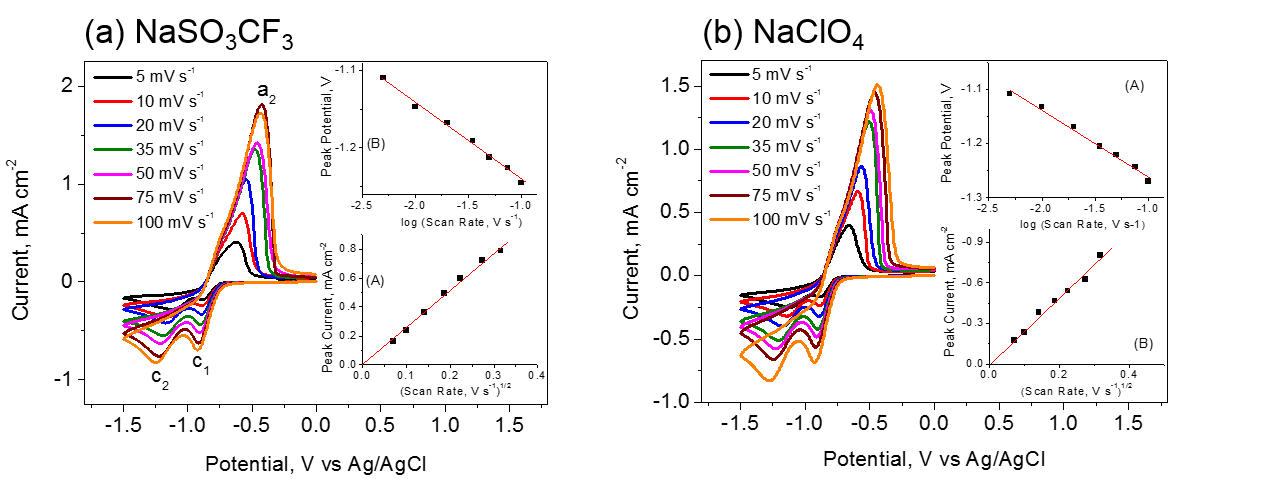


**Figure S7 |** **The second ORR step (peak c_2_) identification.** (**a** and **b**) CVs at various scan rates in 0.1 M NaSO_3_CF_3_ or NaClO4/DMSO. **Insets**: Randles-Sevcik relationship plots of peak currents at c_2_ versus the square root of sweep rates.

**
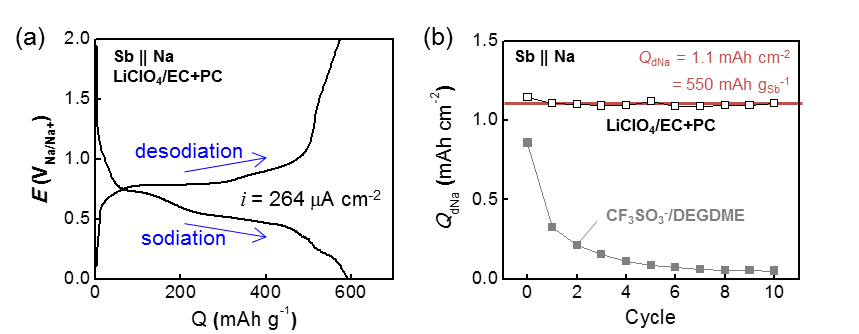
**

**Figure S8 | Pre-lithiating nanoparticular Sb-based electrodes.** Sodium ion battery anode half cells were constructed, where Sb nanoparticles and sodium metal were used as both electrodes in 1 M LiClO_4_ in a mixture of EC and PC as an electrolyte. Pre-sodiated Sb electrodes (Sb:Na) for sodium *ion* oxygen battery cells were prepared by sodiating the electrodes experiencing the pre-cycle followed by the ten stabilization cycles. (**a**) The potential profiles of sodiation followed by desodiation at 264 μA cm^-2^ or 110 mA g^-1^ after the SEI layer is formed at 158.4 μA cm^-2^ or 66 mA g^-1^ in the initial sodiation-desodiation cycle (pre-cycle). (**b**) The desodiation capacity (*Q*_dNa_) retention along consecutive ten cycles after the pre-cycle. The CF_3_SO_3_^-^/DEGDME were also used as the electrolyte.


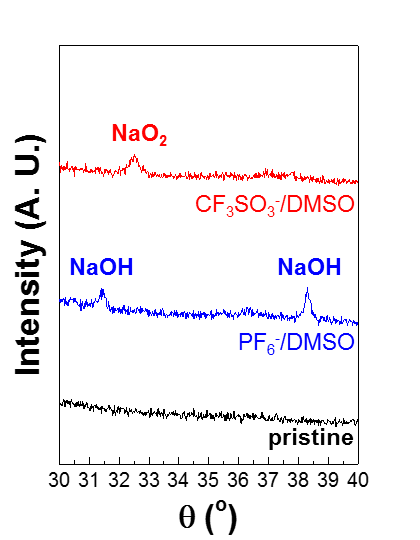


**Figure S9 | X-ray diffraction patterns** of discharge products formed in sodium ion-oxygen cells. The anion/solvent pairs for electrolytes were indicated.

**Supporting References**

(S1) Chang, B.-Y.; Lee, H. J.; Park, S.-M. Mass-Transfer Admittance Voltammetry from Electrochemical Impedance Spectroscopy and Its Applications. *Electroanalysis* **2011**, *23*, 2070-2078.

(S2) Huang, Q.-A.; Park, S.-M. Unified Model for Transient Faradaic Impedance Spectroscopy: Theory and Prediction. *J. Phys. Chem. C* **2012**, *116*, 16939-16950.

(S3) Laoire, C. O.; Mukerjee, S.; Abraham, K. M.; Plichta, E. J.; Hendrickson, M. A. [Influence of Nonaqueous Solvents on the Electrochemistry of Oxygen in the Rechargeable Lithium−Air Battery](http://pubs.acs.org/doi/abs/10.1021/jp102019y). *J. Phys. Chem. C* **2010**, *114*, 9178-9186.

(S4) Liu, W.; Sun, Q.; Yang, Y.; Xie, J.-Y.; Fu, Z.-W. An Enhanced Electrochemical Performance of a Sodium-Air Battery with Graphene Nanosheets as Air Electrode Catalysts. *Chem. Commun.* **2013**, *49*, 1951-1953.
